# The Shutdown of Celiac Disease-Related Gliadin Epitopes in Bread Wheat by RNAi Provides Flours with Increased Stability and Better Tolerance to Over-Mixing

**DOI:** 10.1371/journal.pone.0091931

**Published:** 2014-03-14

**Authors:** Javier Gil-Humanes, Fernando Pistón, Francisco Barro, Cristina M. Rosell

**Affiliations:** 1 Instituto de Agricultura Sostenible, CSIC, Córdoba, Spain; 2 Instituto de Agroquímica y Tecnología de Alimentos, CSIC, Valencia, Spain; Centro di Riferimento Oncologico, IRCCS National Cancer Institute, Italy

## Abstract

Celiac disease is a food-sensitive enteropathy triggered by the ingestion of wheat gluten proteins and related proteins from barley, rye, and some varieties of oat. There are no interventional therapies and the only solution is a lifelong gluten-free diet. The down-regulation of gliadins by RNAi provides wheat lines with all the gliadin fractions strongly down-regulated (*low-gliadin)*. The technological properties of doughs prepared from the *low-gliadin* lines indicated a general weakening effect, although some of the lines displayed similar properties to that of the wild-type lines. In contrast, the stability was increased significantly in some of the transgenic lines, indicating better tolerance to over-mixing. Results reported here are the first analyses of the mixing and bread-making quality of the wheat lines with all gliadin fractions strongly down-regulated. Flour from these lines may be an important breakthrough in the development of new products for the celiac community. These lines might be used directly or blended with other non-toxic cereals, as raw material for developing food products that can be safely tolerated by CD patients and others with gluten intolerance or gluten sensitivity, incrementing the range of available food products and enhancing their diet.

## Introduction

Gliadin proteins of wheat gluten are the main players that cause celiac disease (CD), a food-sensitive enteropathy that occurs in genetically predisposed individuals upon ingestion of wheat gluten proteins and similar proteins from barley and rye [1]–[3]. The disease occurs almost worldwide but the prevalence is particularly high in Western countries (1%) [4], [5]. CD is characterized by small intestinal inflammation, villous atrophy and crypt hyperplasia, and the only treatment available is a lifelong gluten-free diet. However, adhering to a strict gluten free diet is challenging as gluten is a ubiquitous additive in various foods such as sausages, soups, instant coffees, sauces (mustard, soy sauce, gravy powder, syrup…), and also other products such as some pharmaceutical products, lipsticks and toothpastes. Gluten proteins are also major determinants of technological properties of wheat as their unique biomechanical properties allow wheat flour to be processed into leavened bread, pasta and noodles [6], [7].

Gluten proteins account for around 80% of the total grain proteins [6] and they are classified into two families: glutenins and gliadins. The glutenins comprise the high molecular weight (HMW) and low molecular weight (LMW) fractions, forming complex polymers related with dough elasticity. The gliadins comprise three structural types α-, γ- and ω-gliadins [8] fractions, which are monomeric components and contribute to the extensibility and viscosity of the dough [9]. The development of wheat varieties with reduced content of CD-related epitopes would be extremely important for CD patients to improve their diet, and would even help to reduce the CD incidence, as it is also observed that the initiation of CD is associated with the level and duration of exposure to gluten [10], [11]. However, the development of such wheat varieties is not an easy task. Although a number of CD-related epitopes are derived from glutenins [12], the majority of these epitopes reside in the gliadin fraction [13], [14]. Gliadin genes are located on three chromosomes in bread wheat, with a variable number of copies of the genes within the same gliadin family and also the number of stimulatory epitopes present in each gene can vary [15], [16].

In previous works RNA interference (RNAi)-mediated gene silencing was used to down-regulate the content of the γ-gliadins [17], and all the α-, γ- and ω-gliadins [18] in bread wheat. The last have been demonstrated to have low stimulatory capacity of T-cells isolated from intestinal CD lesions and therefore, they could potentially be used, directly or blended, as raw material for foodstuff tolerated by CD patients. The effect of the down-regulation of γ-gliadins on the bread-making quality was evaluated in the transgenic lines of cv. ‘Bobwhite’ (BW208 and BW2003) by using the Mixolab and the sodium dodecyl sulfate sedimentation (SDSS) test [19], and also in three commercial lines of bread wheat with the Mixograph and the SDSS test [20]. Gil-Humanes *et al.* [21] studied the effect of the down-regulation of γ-gliadins, and all the α-, γ- and ω-gliadins on the morphology of the protein bodies of the endosperm, and Pistón *et al*. [22] characterized the effect of different combinations of promoters, inverted repeats, and genotype on the level of down-regulation of gliadins. However, the effect of silencing all the α-, γ- and ω-gliadins on the rheological and functional properties of bread wheat has not been assessed so far. Gliadins have generally been associated with negative effects on dough properties and bread-making quality [23]–[25], resulting in a decrease of dough strength when added to wheat flour [26], [27]. However, gliadins have been also related with the formation of extensive gluten film networks through covalent and non-covalent bonding with other gluten proteins, and consequently may play an important role in the bread-making quality by improving gas retention and loaf volume [27].

In the present study we report the technological properties of twenty bread wheat lines with down-regulation of all gliadins by RNAi. These lines are important for elaborating wheat flour-derived products with low content of CD-related epitopes. They are also important for understanding the role of the gliadins and LMW-glutenins on the bread-making quality of wheat.

## Materials and Methods

### Plant Material

Thirteen transgenic lines of *Triticum aestivum* cv ‘Bobwhite 208’ (BW208) and seven transgenic lines of *T. aestivum* cv ‘Bobwhite 2003’ (BW2003) with reduced levels of gliadins, and the corresponding wild-type lines were used in this study. Both wild types derive from the original cross CM 33203 with the pedigree Aurora//Kalyan/Bluebird/3/Woodpecker made by the CIMMYT bread wheat program in the early 1970s, and from which 129 wheat sister lines (most of them containing the T1BL.1RS translocation from rye) were obtained. BW208 derives from the SH 98 26 ‘Bobwhite’ line described as highly transformable by Pellegrineschi *et al.* [28], and does not contain the rye translocation. On the other hand, cultivar BW2003 was also selected for its high transformation efficiency, and was found to carry the T1BL.1RS translocation. All transgenic lines were previously reported or obtained as described by Gil-Humanes *et al.* [18], [29] using four hairpin RNA (hpRNA) vectors: pGhpg8.1 and pGhp-ω/α, which contain an endosperm-specific promoter from a γ-gliadin gene [30] and pghpg8.1 and pDhp-ω/α, with an endosperm-specific promoter from a D-hordein gene [31]. Vectors pGhpg8.1 and pghpg8.1 contained an inverted repeat (IR) sequence from a γ-gliadin gene and were designed to down-regulate the γ-gliadins fraction, whereas pGhp-ω/α and pDhp-ω/α contained an IR sequence encompassing α-, γ- and ω-gliadin genes to target all the gliadin fractions. Lines 28A, 28B, D783, E140 and E146 contained the pDhp-ω/α vector; lines D770, D894 and D793 contained the pGhp-ω/α vector; lines D874 and D876 contained both the pDhp-ω/α and pGDhp-ω/α vectors; lines E33, E35, E39, E42, E76, E82, E83 and E122 contained the pDhp-ω/α and the pghpg8.1; and lines E93 and E96 contained the pGhp-ω/α and the pGhpg8.1 vectors. Transgenic lines were self-pollinated for three to four generations to obtain mostly homozygous plants lines and grains in sufficient quantities for the assays described below.

### Polyacrylamide Gel Electrophoresis (PAGE) Analysis

Mature grains were crushed into a fine powder and used to extract the endosperm storage proteins. Gliadins and glutenins were sequentially extracted, and separated in acidic polyacrylamide gel electrophoresis (A-PAGE) and sodium dodecyl sulfate-PAGE (SDS-PAGE) systems, respectively, as described Gil-Humanes *et al.* [20].

### Mixograph Analysis

Dough mixing properties were determined with a 35 g Mixograph (National Manufacturing Co., Lincoln, NE). Prior to milling, kernel moisture was adjusted to 14% overnight at room temperature with continuous shaking. Milling was carried out in a CyclotecTM 1093 mill (Foss Analytical, Hillerød, Denmark), and then flour was refined through a 250 µm screen. The recommended equation in the AACC 54–40A method [30] to determine the percentage of water absorption (WA) of each sample was optimized for the *low-gliadin* lines and their controls, resulting in the following equation: WA (%) = 52.35+1.5 *Protein (%); where the protein content is expressed on a 14% moisture basis.

### Mixolab Analysis

Mixing and pasting properties of wheat flour samples were studied using the Mixolab analyzer (Chopin Technologies, Villeneuve-la-Garenne Cedex, France). Milling was carried out in a CyclotecTM 1093 mill (Foss Analytical, Hillerød, Denmark), as described above. For each sample 50 g of whole meal flour was analyzed with the standard ‘Chopin+’ protocol as described Gil-Humanes *et al.* [19]. The main parameters recorded were the ones initially defined by Rosell *et al.* [32]. Other secondary parameters such as protein weakening range (C2-C1), starch gelatinization range (C3-C2), and the slopes γ and δ were also determined. Three independent replicates of each line were analyzed. The protocol ‘Chopin S’ was used to determine the stability (min) in some of the samples. The ‘Chopin S’ method measures the torque (Nm) during 30 min of mixing at constant speed (80 rpm). Flour from each block was blended proportionally and a single measurement of stability was carried out for each sample due to the limited amount of remaining flour.

### Seed Protein and Starch, and SDSS Test

Thousand kernel weight (g) was determined using 1000 seeds from each sample. Test weight (g l^−1^) was calculated by weighing 100 ml of cleaned grains from each sample. The protein content of whole flour was calculated from the Kjeldahl nitrogen content (%N×5.7) according to the standard ICC method no. 105/2 [33], and the starch content was determined according to the standard ICC method no. 123/1 [34]. Both parameters were expressed on a 14% moisture basis. Gliadins and LMW glutenins quantification for calculation of the gliadin/LMW ratio was made by reversed-phase high-performance liquid chromatography (RP-HPLC) as described by Pistón *et al.* [35]. The SDS sedimentation volume was determined as described by Williams *et al.* [36]. Two or three technical replicates were carried out for each biological sample.

### Experimental Design and Statistical Analysis

The homozygous transgenic lines were assayed on samples produced in two consecutive growing seasons (2010 and 2011) using randomized complete block designs with three replicates of five plants each. Data were analyzed with the statistical software R version 2.12.1 [37] using the Graphical User Interface (GUI) R Commander. The randomized design was generated with the package *agricolae* (function *design.rcbd*). Major assumptions of analysis of variance (ANOVA) were confirmed by the Shapiro-Wilk’s test for normal distribution (function *shapiro.test*, package *stats*), by the Levene’s test for homogeneity of variances (function *leveneTest*, package *car*), and by the Ramsey regression equation specification error test (RESET) for linearity (function *resettest*, package *lmtest*); and variables were transformed if required. The differences in the data were assessed using analysis of the variance (ANOVA) (function *aov*, package *agricolae*), followed by the two-tailed Dunnett’s *post hoc* test for median multiple comparisons. *P* values lower than 0.05 were considered significant, and lower than 0.01 were considered highly significant. Principal component analysis (PCA) was carried out for multivariate statistical analysis of SDSS volume and Mixolab parameters by using the SPSS version 11.0 statistical software package (SPSS Inc., Somers, NY). Varimax rotation was applied to extract the principal components.

## Results and Discussion

### A-PAGE and SDS-PAGE Analyses

**Figure 1:**
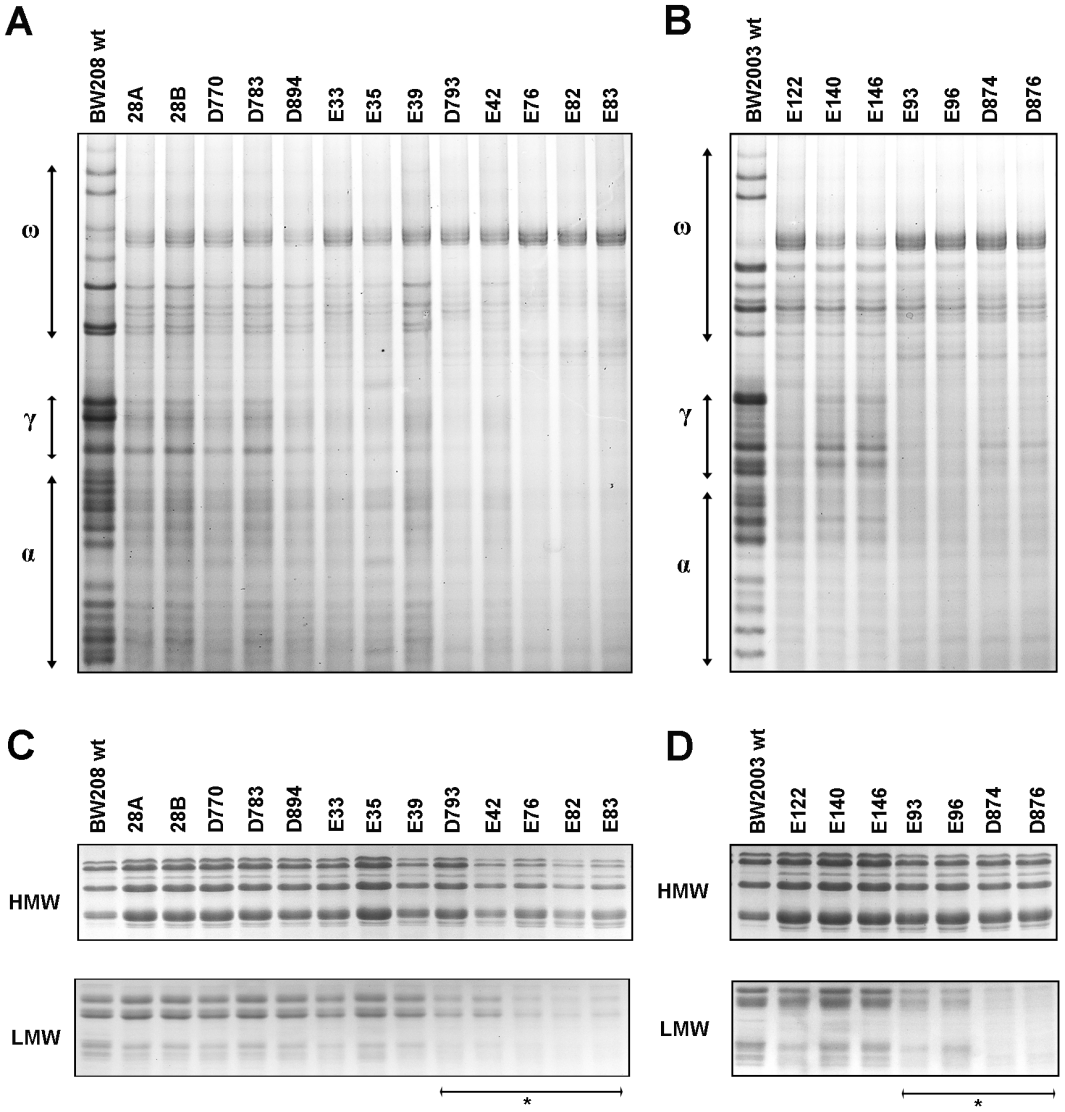
PAGE-protein profiles. A-PAGE of gliadins from (A) BW208 and (B) BW2003 genotypes; and SDS-PAGE of glutenins from (C) BW208 and (D) BW2003 genotypes. Asterisks indicate *low-LMW* transgenic lines.

In the present study, twenty transgenic lines with reduced content of CD-related epitopes [18] were analyzed to determine the effect that the RNAi-mediated down-regulation of gliadins has on the bread-making quality. The A-PAGE gel (Fig. 1A and 1B) confirmed the effectiveness of the down-regulation of the gliadin fraction after 3–4 generations of self-pollination. The bands corresponding to the HMWs were more intense in most of the transgenic lines of both background wheat genotypes than in the non-transgenic wild types (Fig. 1C and 1D), indicating the over-expression of this fraction as reported previously Gil-Humanes *et al.* [18], [21]. The LMW fraction was reduced in nine of the transgenic lines, possibly due to high homology between the LMW mRNA sequences and the siRNA sequences formed from the hpRNA structures (Fig. 1C and 1D). This LMW silencing is stable since it has been observed in several consecutive generations, and is independent of the hairpin RNA (hpRNA) construct used. This phenomenon allows us to classify the *low-gliadin* lines into two groups: *high-LMW* (lines 28A, 28B, D770, D783, D894, E33, E35, E39, E122, E140, and E146), with a LMW content comparable to the wild types; and *low-LMW* (lines E42, D793, E76, E82, E83, E93, E96, D874 and D876), with a reduced LMW content in comparison to the wild types.

### Mixing and Pasting Properties of the Dough

**Figure 2:**
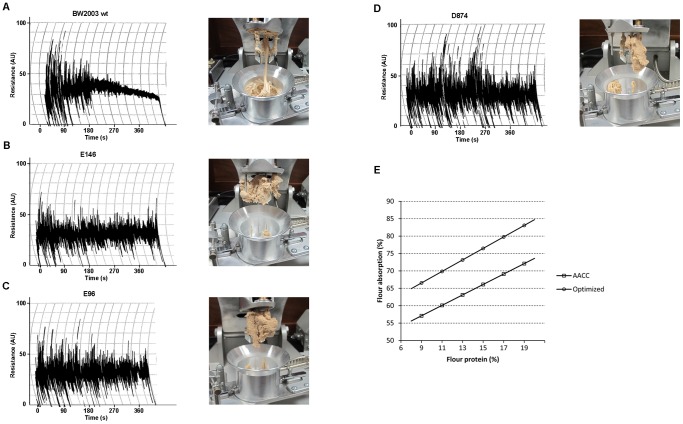
Mixing properties of the BW2003 lines. Mixograms (A–D) of the doughs from the wild type and the transgenic lines of the BW2003 genotype, and (E) optimized equation for determination of water absorption percentage. Transgenic line E146 is a *high-LMW*, whereas E146 and D784 are *low-LMW* transgenic lines.

**Figure 3:**
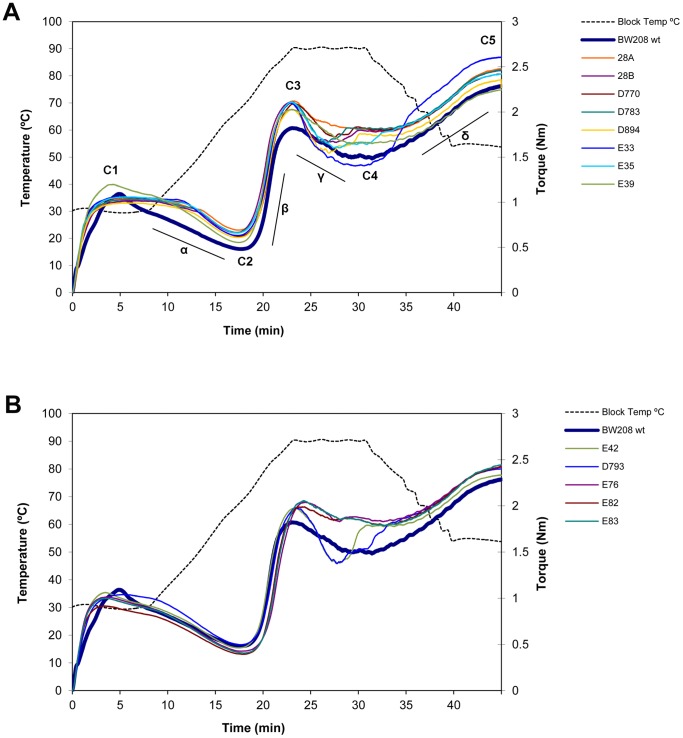
Mixolab curves of transgenic and wild-type lines of genotype BW208. (A) Wild type BW208 and *high-LMW* transgenic lines; (B) wild type BW208 and *low-LMW* transgenic lines. Each curve represents the average of the three blocks of the 2011 assay, obtained with the ‘Chopin+’ method of the Mixolab. The wild type is represented by a wider dark blue line.

The mixing properties of the *low-gliadin* lines were studied with the Mixograph (Figs. 2A–2D, and Fig. S1). The wild types BW208 and BW2003 showed normal mixograms, whereas most of the *low-gliadin* lines showed rheological characteristics unfitted to the standard mixograph profiles, and only three lines namely, E42, D874, and E93 (all *low-LMW*), presented curves similar to the wild types (Fig. S1). A possible explanation for this phenomenon could be the low content of LMW of these lines, or an altered ratio gliadins/LMW (Table S2). However, not all the *low-LMW* lines showed this characteristic, and we did not find any correlation between the gliadins/LMW ratio and the differential behavior in the Mixograph for these lines. Therefore, other factors in the protein network may be influencing the mixing properties of these lines. This altered behavior in most of the transgenic lines impeded the determination of the parameters typically measured with the Mixograph (such as mixing time, peak resistance, peak width, height of the curve at three minutes after the peak, and width of the curve at three minutes after the peak) [35]. In addition, the dough in all the transgenic lines presented a different texture, less extensible and less sticky than the wild-type dough, as observed by manual handling after the mixing process in the Mixograph bowl (Figs. 2A–2D). However, in most of the *low-gliadin* lines the mixograms showed high amplitude (given by the band width) indicating high elasticity [38], and the consistency did not decrease during the mixing, which is associated with higher stability. Therefore, the Mixolab analysis was used to study the rheological properties of doughs from the *low-gliadin* lines (Table 1, Table S1, Fig. 3 and Fig. S2). The Mixolab simulates the conditions of the baking process, allowing the analysis of the physico-chemical properties of dough by recording the mechanical changes under controlled mixing and temperature constraints [32]. The Mixolab has been successfully compared with traditional methods for determining dough quality like Farinograph, Extensograph, Amylograph, Falling Number and Rapid Visco-Analyzer (RVA) [39]–[42], and has previously been used to determine the quality of transgenic wheat lines with reduced content of γ-gliadins [19]. During the first stage of the Mixolab, water absorption and both quantitative and qualitative protein content are of great importance in the formation of the viscoelastic network [7], and the mechanical energy input supplied during mixing of flour is recorded. Development time, C1 and stability are highly correlated with dough strength and protein quality (the stronger the dough, the longer the development time and the stability, and the higher the C1), although this correlation might be altered by the proportion of the different protein fractions that determines the formation of intra- and inter-molecular protein bonds [7]. Most of the transgenic lines reported here showed low values of development time, indicating a weakening effect of the dough. In addition, in most of the BW208 transgenic lines C1 was significantly reduced, showing also a decrease in dough strength (Table 1). Gliadins have been reported to decrease the overall dough strength when added to base flours [24], [26], [43], [44]; conversely, our results indicate that the reduction of the gliadin content has a negative effect on dough strength, suggesting an important role of the gliadins in the dough strength. Fido *et al.* [27] reported that gliadins may be involved in the formation of extensive gluten film networks through covalent and non-covalent bonding with other gluten proteins. In addition, Kasarda [45] suggested that gliadins having an odd number of cysteine residues might be able to form inter-molecular disulfide bonds. Our results agree with the proposed by Fido *et al.* [27] and show that gliadins may play an important role in the formation of the three dimensional viscoelastic structure with gas-retaining properties, positively contributing to the dough strength and the bread-making quality.

**Table 1:** Mixolab parameters of flour samples.

Line	Developmenttime (min)	Amplitude(Nm)	Maximum torque(C1) (Nm)	Minimum torque(C2) (Nm)	Temperature atC2 (°C)	α (Nm/min)	β (Nm/min)	C3(Nm)	C4(Nm)	C5(Nm)	Cooking stability(C3–C4) (Nm)	Setback (C5–C4)(Nm)
**BW208 wt**	4.3	0.07	1.18	0.51	57.43	−0.05	0.55	1.85	1.51	2.38	0.51	0.86
28A	3.3**	0.09	1.08**	0.65***	57.15	−0.07***	0.55	2.09***	1.60	2.23	0.51	0.64
28B	3.2	0.08	1.05***	0.65***	57.32	−0.07***	0.49	2.07***	1.57	2.28	0.61	0.72
D770	2.8***	0.08	1.09	0.61***	57.17	−0.08***	0.51	2.05***	1.64	2.30	0.60	0.66
D783	2.9***	0.08	1.07***	0.65***	57.43	−0.08***	0.54	2.09***	1.67	2.39	0.65	0.72
D894	2.7***	0.08**	1.07	0.60***	56.75	−0.07***	0.49	2.04***	1.55	2.35	0.66	0.81
E33	2.8***	0.07	1.09**	0.59***	57.70	−0.08***	0.56	2.07***	1.54	2.55	0.78**	1.02
E35	2.9***	0.08	1.10	0.62***	58.55	−0.08***	0.58	2.10***	1.68	2.47	0.74	0.79
E39	2.8***	0.07	1.15	0.49	58.58	−0.06	0.65**	1.97***	1.73	2.42	0.66	0.69
D793	3.1***	0.06	1.08**	0.48	57.65	−0.06	0.54	1.94***	1.55	2.40	0.77	0.86
E42	2.4***	0.07	1.08**	0.44	57.95	−0.06	0.62	1.95***	1.54	2.37	0.75	0.83
E76	2.3***	0.05	0.97***	0.41***	59.12**	−0.06	0.60	2.02***	1.75	2.40	0.50	0.64**
E82	2.0***	0.05***	0.90***	0.37***	58.77	−0.05	0.60	1.95***	1.70	2.31	0.53	0.61***
E83	2.1***	0.05	1.04***	0.42***	58.57	−0.06	0.53	2.03***	1.85***	2.52	0.67	0.67
**Av. transgenics**	2.7***	0.07	1.06***	0.54	57.88	−0.07***	0.56	2.03***	1.64	2.38	0.65	0.74
**BW2003 wt**	4.4	0.07	1.17	0.47	58.82	−0.06	0.48	1.67	1.37	2.01	0.43	0.64
E122	3.0***	0.08	1.22	0.47	61.18***	−0.08***	0.40***	1.56	0.74***	1.00***	0.68	0.26***
E140	4.0	0.07	1.12	0.55***	59.33	−0.09***	0.50	1.88***	1.32	1.75	0.66	0.43***
E146	3.5	0.08**	1.13	0.60***	58.28	−0.08***	0.45	1.84**	1.20	1.61**	0.62	0.41***
E93	2.6***	0.06	1.11	0.38***	61.08***	−0.06	0.44	1.51**	0.64***	0.89***	0.65	0.25***
E96	2.3***	0.07	1.14	0.42**	60.22**	−0.07	0.44	1.52**	0.64***	0.90***	0.55	0.26***
D874	2.4***	0.07	1.10**	0.41**	60.70***	−0.06	0.44	1.56	0.74***	1.03***	0.67	0.30***
D876	2.4***	0.07	1.15	0.44	59.70	−0.07***	0.41**	1.54	0.73***	1.02***	0.63	0.29***
**Av. transgenics**	2.9***	0.07	1.14	0.47	60.07***	−0.07***	0.44**	1.64	0.86**	1.18***	0.64***	0.32***

Average values obtained in the 2010 and 2011 assays are shown for each transgenic and wild-type line. Means are significantly different to control as determined by Dunnett’s multiple comparison as follows: *P<0.1; **P<0.05; ***P<0.01.

After the first stage of mixing, the combination of mechanical stress and increased temperature produces protein destabilization, unfolding, and aggregation. It is normally accepted that the greater the decrease of consistency, the lower the protein quality. In this phase, the parameters measured were the slope α (that represents the speed of protein weakening due to heating), C2, and the protein weakening range (C2-C1) [32], [46]. We observed a greater slope α in the *high-LMW* transgenic lines than in the wild types, but not in the *low-LMW* lines (Table 1). However, the protein weakening range was significantly reduced in most of the BW208 transgenic lines, and in the *high-LMW* BW2003 transgenic lines, indicating more stable doughs during heating.

The second part of the Mixolab curve represents the dough viscosity and is mainly influenced by the starch properties. This part can be divided in three phases: starch gelatinization, amylolytic activity, and starch retrogradation. The slope β (or starching speed of the dough) and C3 represent the starch gelatinization of the dough. During this stage, starch granules swell and absorb water, and amylose molecules leach out resulting in an increase of the viscosity. Gelatinization was very similar in the transgenic lines with respect to the wild types, with the slope β being unaffected in most of the cases, and C3 only increased in the BW208 transgenic lines, indicating little change in the gelatinization of the dough.

The amylolytic activity is mainly determined by the parameters slope γ, C4 and cooking stability (C3–C4). The intensity of the decrease of the consistency depends on amylase activity. Only the transgenic lines of BW2003 showed altered amylolytic activity with respect to the wild type, with an increased slope γ, decreased values of C4, and decreased cooking stability (higher values of C3–C4) (Table 1 and Table S1). On the other hand the starch retrogradation is mainly represented by the slope δ, C5 and setback (C5–C4). At this stage, the decrease of temperature provokes an increase in the consistency due to gel formation associated to amylose re-crystallization. In our study the retrogradation was affected in BW2003 transgenic lines, showing reduced values of δ, C5 and setback (Table 1 and Table S1). The altered amylolytic activity and retrogradation in BW2003 lines indicate a different behavior of the starch in the heating process. A previous study, Gil-Humanes *et al.* [19], reported a similar effect in the starch properties of transgenic wheat lines with RNAi-mediated down-regulation of γ-gliadins. In that study, a negative correlation was observed between the Mixolab parameters describing the starch behavior (C3, C4 and C5) and both the content of glutenins and the ratio glutenins:gliadins. Consequently, the down-regulation of gliadins, and subsequent increase of the ratio glutenins:gliadins, may have provoked the observed alteration of the starch properties in the BW2003 transgenic lines. Furthermore, in the late phase of the Mixolab, most of the BW208 transgenic lines of the 2011 assay showed the formation of a bump in the in the curve, between the C3 and C5 points (Fig. 3A and 3B). This phenomenon has been previously reported in amylograms during the cooling stage [47], [48]. The bump area has been related with the extent of the formation of amylose-lipid complex during the cooling phase. High bump areas are associated to high concentration of amylose-lipid complex, and it is connected to a softened effect on the bread crumb [47].

**Figure 4:**
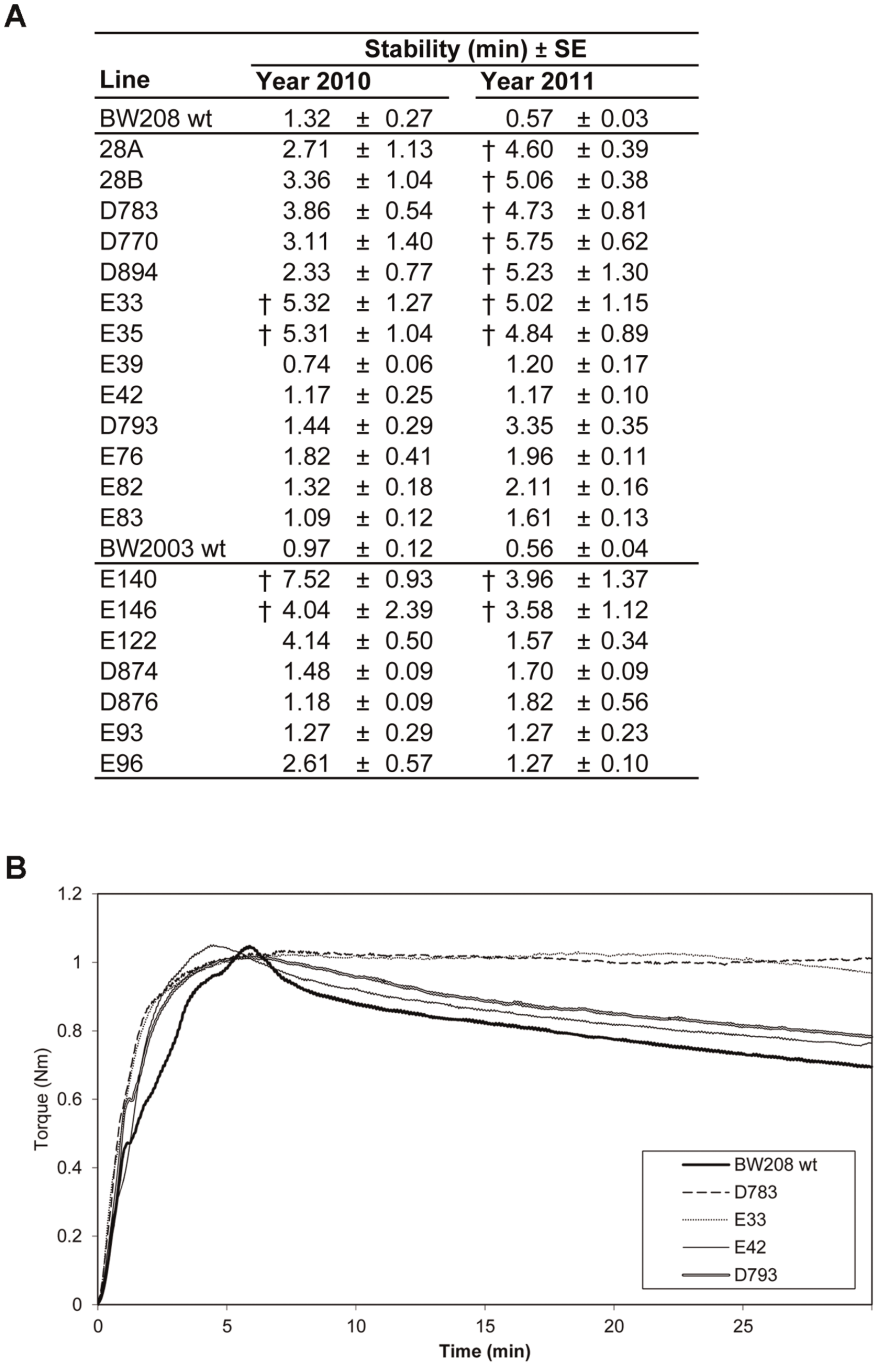
Stability of wild types and transgenic lines obtained with the ‘Chopin +’ and ‘Chopin S’ methods. (A) Stability of transgenic and wild type lines (assays 2010 and 2011) determined with the ‘Chopin +’ method. †, indicate the samples with stability values that exceeded the first 8 minutes of mixing with the ‘Chopin +’ method. (B) Stability of wild type BW208, *high-LMW* lines D783 and E33, and *low-LMW* lines E42 and D793 as determined by the ‘Chopin S’ method.

The stability, or elapsed time at which the torque is maintained constant after C1 (torque ≥98% C1), was calculated in all the lines with the program ‘Chopin +’ of the Mixolab (Fig. 4A). Some of the transgenic lines (all *high-LMW* lines) had high stability values, with the torque remaining constantly above 98% of C1 after the first 8 minutes of the mixing, moment at which the temperature starts to rise from 30°C to 90°C in the ‘Chopin +’ program. In order to calculate the real stability of these lines, as determined by the protein weakening due to the mixing effect, we carried out the ‘Chopin S’ program of the Mixolab, consisting in 30 min of mixing at a constant temperature of 30°C. It is notable that doughs in the *high-LMW* lines were stable after 30 minutes of mixing (Fig. 4B), indicating an increased resistance to over-mixing. The main difference between these high stable lines and the rest of transgenic lines is a higher content of LMW. In general, the LMW glutenins have been associated with dough resistance and extensibility [49], [50], and have been reported to influence the formation of the gluten polymer due to the presence of cysteine residues available for inter-molecular disulfide bonds (reviewed by [51]). Consequently, the LMW glutenins might be playing an important role in the dough stability of the *low-gliadin* wheat lines.

### Grain Quality and Composition

Thousand kernel weight and grain test weight were significantly reduced in most of the *low-LMW* transgenic lines, whereas the starch and protein contents were not significantly affected in most of the BW208 and BW2003 transgenic lines (Table S2). All the *low-LMW* lines had reduced values in the SDS sedimentation test, whereas the *high-LMW* lines showed sedimentation volumes comparable to the wild types. The SDSS test is typically used in bread wheat breeding programs to predict gluten strength and baking quality [52]. The sedimentation in the SDS test is the result of the swelling of the glutenin strands [53], and high volumes of SDSS are typically associated with stronger gluten and superior bread-making quality [54], [55]. Therefore, the SDSS test also confirmed that the *high-LMW* transgenic lines have comparable bread-making properties to that of the wild-type lines, and higher than the *low-LMW* lines. Similar results of the SDSS test were obtained previously for some of these lines [18], with most of the transgenic lines showing comparable volumes of SDSS than the wild types. These results fit with the obtained with the ‘Chopin +’ program of the Mixolab, and confirm the importance of the LMW in the formation of the gluten polymer, and consequently the influence on the gluten strength and the bread-making quality of the *low-gliadin* transgenic lines.

### Principal Components Analysis (PCA)

**Figure 5:**
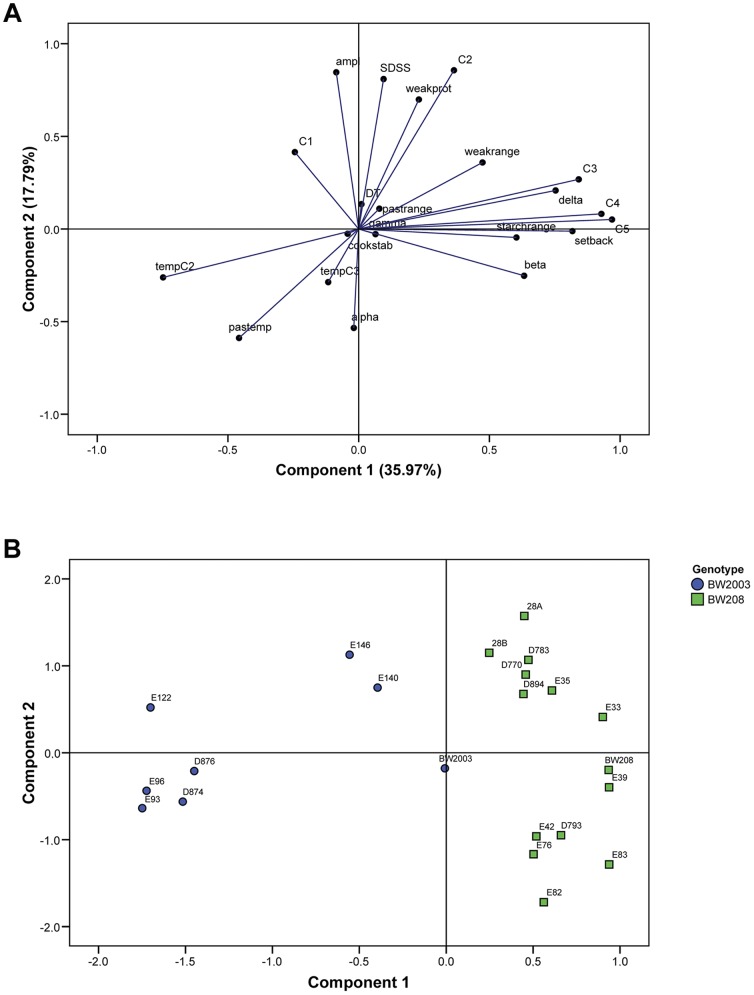
Principal component analysis (PCA) of the technological quality parameters studied. (A) Correlation loadings plot from principal component analysis showing the Mixolab and SDSS test parameters. The eigenvalues of the correlation matrix are symbolized as vectors representing traits that influence the principal components 1 and 2; (B) Score plot for the principal components 1 and 2 of the transgenic and wild-type lines assayed with the Mixolab and the SDSS test.

In order to reduce the complexity and the number of variables reported in the present work, we carried out a reduction of the variables by PCA of the Mixolab parameters and SDSS test. The result showed that the first two components of the PCA explained 53.75% of the total variance. The first component, which represented 35.97% of the variability, was mainly determined by the starch properties of the dough, with the starch retrogradation parameters (C4, C5 and setback) being the most important (Fig. 5). Collar [56] reported a high correlation of the viscosity during cooking and cooling as determined with the RVA with delayed bread staling and high sensory scores of fresh bread. RVA and Mixolab were reported to be highly correlated [39], [57]. Thus, component 1 of the PCA reported in the present work might be used as a predictor of good sensory quality and bread-firming behavior during storage. The second component (that explained 17.79% of the variance) was related with protein quality since it was mostly influenced by SDSS, amplitude, and C2 (Fig. 5). High SDSS volumes have been correlated with stronger gluten and superior bread-making quality [54], [55], whereas the amplitude in the Mixolab curve has been defined as an indicative of the extensional properties of the dough during mixing [38]. Therefore, although one of the main indicators of gluten strength, development time, is not correlated with component 2 of the PCA, we can assume that the bread-making quality and gluten strength are positively associated to high values of the component 2 of the PCA analysis. BW208 and BW2003 wild types were found to be very close with respect to the second component but separated with respect to the first component, indicating that the differences between them might be mainly explained by differences in the starch behavior. However, the transgenic lines of BW208 and BW2003 showed a different response respect to their respective wild types (Fig. 5). In BW208 two groups were formed respect to the second component. In the first, the *high-LMW* lines 28A, 28B, D770, D783, D894, and E35 grouped together with higher values of the component 2 than the wild type, indicating a higher level of protein quality. In the second, the *low-LMW* lines E42, D793, E76, E82, and E83 formed another group with lower values of the component 2, and consequently lower quality of dough proteins. Lines E33 and E39 were separated from the groups described above and were found closer to the wild type BW208. Similarly, the BW2003 *low-LMW* lines E93, E96, D874 and D876 grouped together, with lower values for both components 1 and 2 than the group formed by the *high-LMW* lines E140 and E146 (Fig. 5B). In general, the *high-LMW* of each genotype grouped very close in the PCA, showing greater values of principal components 1 and/or 2, and consequently higher quality than their *low-LMW* counterparts (Fig. 5B). This indicates the importance of the LMW glutenins on the mixing and pasting properties of the dough.

## Conclusions

In the present work, the bread-making quality of twenty transgenic lines with reduced content of CD-related epitopes has been analyzed, representing a predictive qualitative analysis of these wheat lines. The Mixolab showed a weakening effect of the dough in the transgenic lines. However, the stability of the *high-LMW* lines was increased, indicating improved tolerance to over-mixing. In addition, the *high-LMW* transgenic lines also showed similar bread-making quality to that of the wild-type lines as determined by the SDSS test. Overall, the *high-LMW* transgenic lines of BW208 showed higher mixing and bread-making quality. These *high-LMW* lines might be used, directly or blended with other non-toxic cereals, as raw material for developing foodstuff tolerated by CD patients and other gluten intolerants. The results presented here indicate that the quality of these products would be very similar to other gluten containing baked goods.

## Supporting Information

Figure S1 **Mixograms of the doughs from wild type and transgenic lines of the BW208 and BW2003 genotypes.**
(TIF)

Figure S2 **Mixolab curves of transgenic and wild-type lines of genotype BW2003.** (A) Wild type BW2003 and *high-LMW* transgenic lines; (B) wild type BW2003 and *low-LMW* transgenic lines. Each curve represents the average of the three blocks of the 2011 assay, obtained with the ‘Chopin+’ method of the Mixolab. The wild type is represented by a wider dark blue line.
(TIF)

Table S1 **Secondary Mixolab parameters of the flour samples.** Average values obtained in the 2010 and 2011 assays are shown for each transgenic and wild-type line. Means are significantly different to control as determined by Dunnett’s multiple comparison as follows: *P<0.1; **P<0.05; ***P<0.01.
(DOC)

Table S2 **Seeds characteristics and composition, and SDSS test.** Gli/LMW, ratio gliadins/LMW glutenins. Means are significantly different to control as determined by Dunnett’s multiple comparison as follows: *P<0.1; **P<0.05; ***P<0.01.
(DOC)
