# Supplementary figures and images for: The Shutdown of Celiac Disease-Related Gliadin Epitopes in Bread Wheat by RNAi Provides Flours with Increased Stability and Better Tolerance to Over-Mixing
Source: PLoS One. 2014 Mar 14;9(3):e91931. doi: 10.1371/journal.pone.0091931 (PMC3954839; doi:10.1371/journal.pone.0091931)

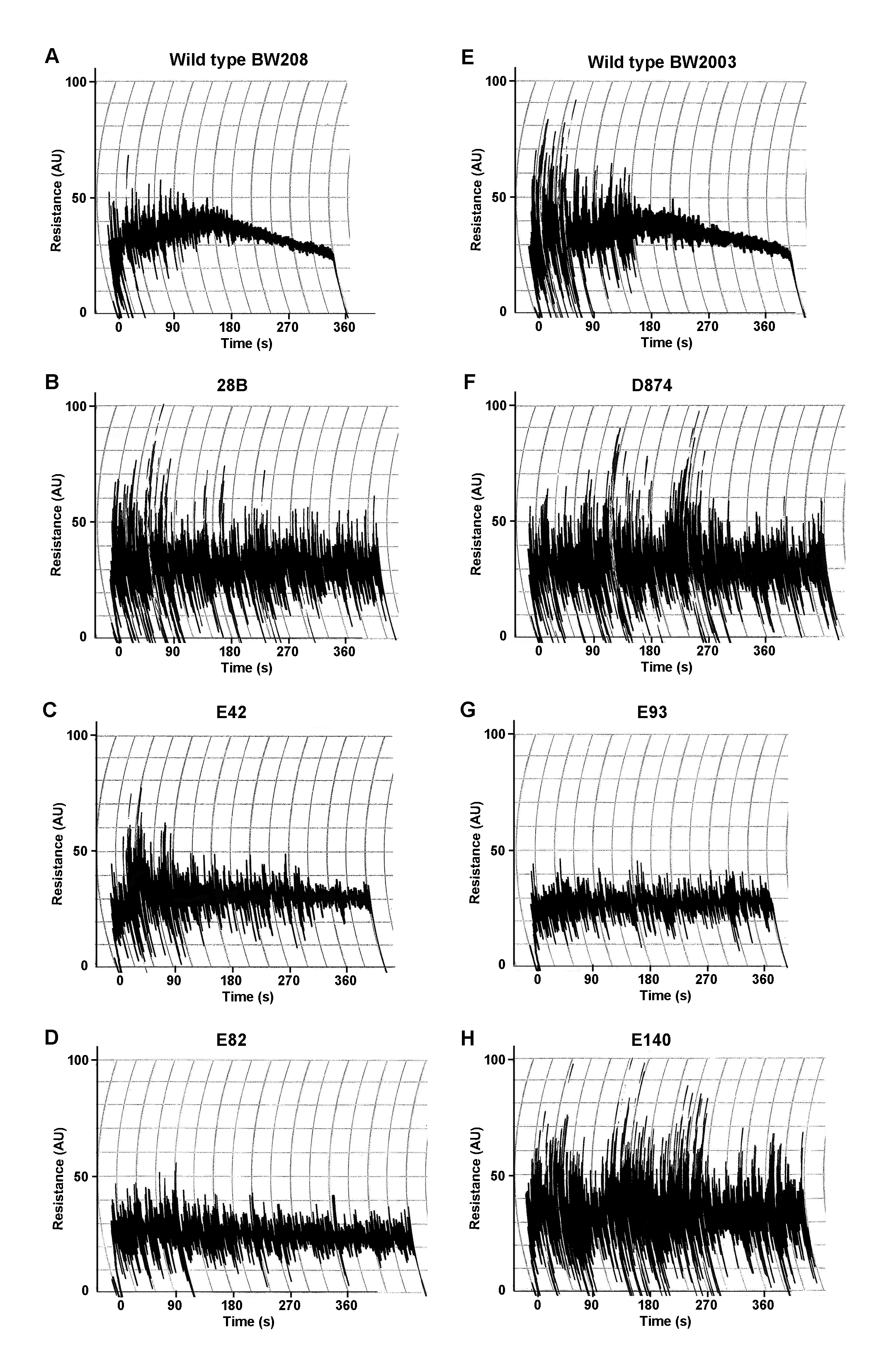

Supplement: Figure S1 — Mixograms of the doughs from wild type and transgenic lines of the BW208 and BW2003 genotypes. (TIF) [file pone.0091931.s001.tif]

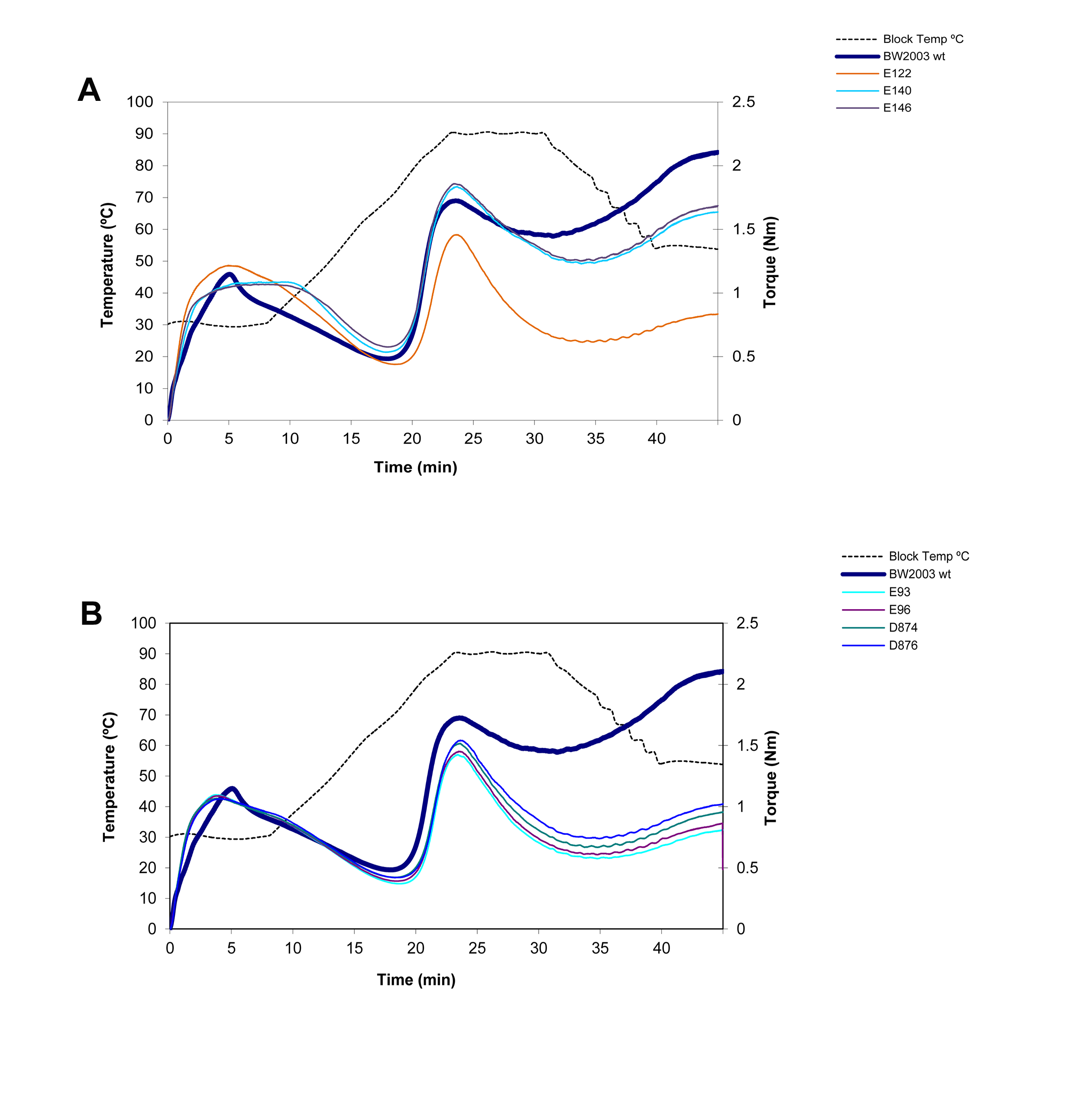

Supplement: Figure S2 — Mixolab curves of transgenic and wild-type lines of genotype BW2003. (A) Wild type BW2003 and high-LMW transgenic lines; (B) wild type BW2003 and low-LMW transgenic lines. Each curve represents the average of the three blocks of the 2011 assay, obtained with the ‘Chopin+’ method of the Mixolab. The wild type is represented by a wider dark blue line. (TIF) [file pone.0091931.s002.tif]
