# Supplementary material for: The Shutdown of Celiac Disease-Related Gliadin Epitopes in Bread Wheat by RNAi Provides Flours with Increased Stability and Better Tolerance to Over-Mixing
Source: PLoS One. 2014 Mar 14;9(3):e91931. doi: 10.1371/journal.pone.0091931 (PMC3954839; doi:10.1371/journal.pone.0091931)
Supplement: Table S1 — Secondary Mixolab parameters of the flour samples. Average values obtained in the 2010 and 2011 assays are shown for each transgenic and wild-type line. Means are significantly different to control as determined by Dunnett’s multiple comparison as follows: *P<0.1; **P<0.05; ***P<0.01. (DOC) [file pone.0091931.s003.doc]

| **Line** | **Beginning protein weakening** (ºC) | | **Initial pasting temp.** (ºC) | | **Temp at C3** (ºC) | | **Protein weakening range** (C2-C1) (Nm) | | **Starch gelatinitation range** (C3-C2) (Nm) | | **Pasting temp. range** (ºC) | | **γ** (Nm/min) | | **δ** (Nm/min) | |
| --- | --- | --- | --- | --- | --- | --- | --- | --- | --- | --- | --- | --- | --- | --- | --- | --- |
| **BW208 wt** | 35.6 |  | 63.0 |  | 78.8 |  | -0.66 |  | 1.33 |  | 21.4 |  | -0.06 |  | 0.07 |  |
| 28A | 40.8 | ******* | 62.9 |  | 79.1 |  | **-0.43** | ******* | **1.43** | ******* | 22.0 |  | -0.09 |  | 0.07 |  |
| 28B | 41.9 | ******* | 62.3 |  | 79.7 |  | **-0.41** | ******* | **1.43** | ******* | 22.4 |  | -0.11 |  | 0.07 |  |
| D770 | 39.9 | ******* | 62.3 |  | 79.4 |  | **-0.48** | ******* | **1.44** | ******* | 22.2 |  | -0.10 |  | 0.06 |  |
| D783 | 41.2 | ******* | 62.3 |  | 79.0 |  | **-0.42** | ******* | **1.45** | ******* | 21.6 |  | -0.11 |  | 0.06 |  |
| D894 | 40.2 | ******* | 61.9 |  | 79.1 |  | **-0.47** | ******* | **1.44** | ******* | 22.3 |  | -0.12 |  | 0.06 |  |
| E33 | 39.5 | ******* | 63.0 |  | 79.4 |  | **-0.50** | ******* | **1.48** | ******* | 21.7 |  | -0.11 |  | 0.09 |  |
| E35 | 40.0 | ******* | 63.5 |  | 80.2 |  | **-0.49** | ******* | **1.48** | ******* | 21.6 |  | -0.07 |  | 0.07 |  |
| E39 | 36.3 |  | 64.1 |  | 79.9 |  | -0.67 |  | **1.49** | ******* | 21.3 |  | -0.05 |  | 0.07 |  |
| D793 | 35.7 |  | 63.5 |  | 79.5 |  | **-0.60** | ****** | **1.47** | ******* | 21.9 |  | -0.13 |  | 0.06 |  |
| E42 | 36.2 |  | 64.7 |  | 80.0 |  | -0.64 |  | **1.52** | ******* | 22.1 |  | -0.14 |  | 0.06 |  |
| E76 | 36.1 |  | 66.0 | ******* | 81.4 | ******* | **-0.56** | ****** | **1.61** | ******* | 22.3 |  | -0.07 |  | 0.06 |  |
| E82 | 35.7 |  | 65.6 | ******* | 80.6 | ****** | **-0.53** | ******* | **1.58** | ******* | 21.9 |  | -0.05 |  | 0.05 |  |
| E83 | 35.2 |  | 64.7 | ******* | 80.7 | ****** | -0.62 |  | **1.62** | ******* | 22.2 |  | -0.05 |  | 0.06 |  |
| **Av. transgenics** | 38.4 | ******* | 63.6 |  | 79.8 | ****** | **-0.52** | ******* | **1.49** | ******* | 22.0 |  | -0.09 |  | 0.06 |  |
|  |  |  |  |  |  |  |  |  |  |  |  |  |  |  |  |  |
| **BW2003 wt** | 35.7 |  | 64.2 |  | 80.3 |  | -0.71 |  | 1.21 |  | 21.4 |  | -0.07 |  | 0.06 |  |
| E122 | 36.4 |  | 64.9 |  | 80.4 |  | -0.75 |  | 1.09 |  | **19.2** | ******* | -0.09 |  | **0.03** | ******* |
| E140 | 38.9 | ******* | 63.7 |  | 80.4 |  | **-0.57** | ******* | 1.33 |  | 21.1 |  | -0.07 |  | 0.06 |  |
| E146 | 39.4 | ******* | 63.1 |  | 79.7 |  | **-0.53** | ******* | 1.25 |  | 21.4 |  | -0.08 |  | 0.06 |  |
| E93 | 35.6 |  | 66.4 | ******* | 80.0 |  | -0.73 |  | 1.13 |  | **18.9** | ******* | -0.09 |  | **0.03** | ******* |
| E96 | 35.2 |  | 65.7 |  | 79.8 |  | -0.72 |  | 1.10 |  | **19.6** | ******* | -0.09 |  | **0.03** | ******* |
| D874 | 35.6 |  | 66.2 | ****** | 80.3 |  | -0.69 |  | 1.15 |  | **19.6** | ******* | -0.09 |  | **0.04** | ******* |
| D876 | 35.5 |  | 65.1 |  | 80.0 |  | -0.71 |  | 1.11 |  | 20.3 |  | -0.09 |  | **0.04** | ******* |
| **Av. transgenics** | 36.6 |  | 65.0 |  | 80.1 |  | -0.67 |  | 1.17 |  | **20.0** | ******* | -0.08 | ** | **0.04** | ******* |
