# Supplementary material for: The Shutdown of Celiac Disease-Related Gliadin Epitopes in Bread Wheat by RNAi Provides Flours with Increased Stability and Better Tolerance to Over-Mixing
Source: PLoS One. 2014 Mar 14;9(3):e91931. doi: 10.1371/journal.pone.0091931 (PMC3954839; doi:10.1371/journal.pone.0091931)
Supplement: Table S2 — Seeds characteristics and composition, and SDSS test. Gli/LMW, ratio gliadins/LMW glutenins. Means are significantly different to control as determined by Dunnett’s multiple comparison as follows: *P<0.1; **P<0.05; ***P<0.01. (DOC) [file pone.0091931.s004.doc]

|  | **Year 2010** | | | | | | | | | |  |  | **Year 2011** | | | | | | | | | |  |  |
| --- | --- | --- | --- | --- | --- | --- | --- | --- | --- | --- | --- | --- | --- | --- | --- | --- | --- | --- | --- | --- | --- | --- | --- | --- |
| **Line** | **1000 seeds** (g) | | **Grain test weight** (g l^-1^) | | **SDSS** (ml) | | **Starch** (%) | | **Protein** (%) | | **Gli/ LMW** |  | **1000 seeds** (g) | | **Grain test weight** (g l^-1^) | | **SDSS** (ml) | | **Starch** (%) | | **Protein** (%) | | **Gli/ LMW** |  |
| **BW208 wt** | 44.5 |  | 815.0 |  | 12.9 |  | 52.1 |  | 11.5 |  | 4.9 |  | 44.1 |  | 850.2 |  | 12.7 |  | 53.0 |  | 12.6 |  | 5.1 |  |
| 28A | 41.1 |  | 806.3 |  | 12.4 |  | 53.5 |  | 11.9 |  | 2.4 |  | **37.8** | ******* | 833.1 |  | 12.7 |  | 51.6 |  | 11.8 |  | 0.7 |  |
| 28B | 42.5 |  | 809.0 |  | 12.8 |  | 50.6 |  | 12.0 |  | 1.7 |  | 42.2 |  | 858.6 |  | 12.6 |  | 51.8 |  | 12.1 |  | 0.7 |  |
| D770 | 41.7 |  | 797.0 |  | 12.0 |  | 51.7 |  | 12.1 |  | 1.4 |  | **37.4** | ******* | 822.3 |  | 12.3 |  | 52.6 |  | 13.3 |  | 0.7 |  |
| D783 | 43.4 |  | 820.0 |  | 12.6 |  | 53.1 |  | 12.0 |  | 1.3 |  | 41.7 |  | 836.0 |  | 12.2 |  | 53.3 |  | 12.5 |  | 0.7 |  |
| D894 | 42.0 |  | 801.0 |  | 12.5 |  | 53.0 |  | 12.4 |  | 1.2 |  | 41.1 |  | 842.8 |  | 11.3 |  | 50.9 |  | 12.5 |  | 0.7 |  |
| E33 | 41.4 |  | 790.7 |  | 12.2 |  | 51.4 |  | 13.6 |  | 1.3 |  | 43.1 |  | 842.5 |  | 11.9 |  | 52.4 |  | 13.3 |  | **0.7**** |  |
| E35 | 41.1 |  | 785.5 |  | 12.7 |  | 51.3 |  | 12.6 |  | 1.4 |  | 40.6 |  | 834.3 |  | 11.5 |  | 51.8 |  | 12.5 |  | **0.8**** |  |
| E39 | **37.6** | ******* | **765.6** | ******* | **9.7** | ******* | 50.5 |  | 13.3 |  | 1.9 |  | **38.3** | ****** | 820.2 |  | **10.9** | ******* | 50.1 |  | 13.5 |  | **1.1***** |  |
| D793 | 41.4 |  | **781.7** | ****** | **8.9** | ******* | 52.9 |  | 12.4 |  | 1.6 |  | 39.9 |  | 833.7 |  | **8.0** | ******* | 52.1 |  | 12.5 |  | **0.9***** |  |
| E42 | **36.9** | ******* | 785.3 |  | **9.0** | ******* | 47.3 |  | 12.5 |  | 1.7 |  | **36.1** | ******* | **812.6** | ****** | **9.1** | ******* | 51.1 |  | 13.1 |  | **1.0***** |  |
| E76 | **34.8** | ******* | **759.2** | ******* | **7.0** | ******* | 51.5 |  | 12.6 |  | 2.2 |  | **33.0** | ******* | **801.8** | ******* | **7.3** | ******* | 50.1 |  | 12.8 |  | **0.9**** |  |
| E82 | **35.5** | ******* | **753.7** | ******* | **6.2** | ******* | 48.0 |  | 13.1 |  | 2.8 |  | **36.1** | ******* | **789.7** | ******* | **6.2** | ******* | 53.1 |  | 11.8 |  | **1.2***** |  |
| E83 | **34.8** | ******* | **758.7** | ******* | **6.9** | ******* | 51.0 |  | 13.1 |  | 2.4 |  | **30.5** | ******* | **770.6** | ******* | **6.8** | ******* | **45.8** | ****** | 13.2 |  | **1.5***** |  |
|  |  |  |  |  |  |  |  |  |  |  |  |  |  |  |  |  |  |  |  |  |  |  |  |  |
| **BW2003 wt** | 45.1 |  | 813.4 |  | 12.4 |  | 54.9 |  | 12.1 |  | 4.7 |  | 44.4 |  | 855.2 |  | 11.7 |  | 54.4 |  | 12.0 |  | 4.4 |  |
| E122 | **34.4** | ******* | **772.4** | ******* | 13.1 |  | **49.6** | ****** | 14.9 |  | 2.2 |  | **34.3** | ******* | 817.8 |  | 10.6 |  | **49.9** | ******* | 15.1 |  | 1.4 |  |
| E140 | 43.3 |  | 809.3 |  | 12.8 |  | 54.0 |  | 12.4 |  | **1.9*** |  | 39.8 |  | 830.0 |  | 11.5 |  | 53.5 |  | 12.9 |  | 1.3 |  |
| E146 | 43.5 |  | 812.0 |  | 12.7 |  | 51.3 |  | 13.5 |  | 3.3 |  | 41.5 |  | 851.0 |  | 11.5 |  | 52.7 |  | 12.1 |  | 1.3 |  |
| E93 | **40.7** | ******* | **767.8** | ******* | **9.4** | ******* | 51.6 |  | 13.9 |  | 2.8 |  | **36.6** | ******* | **782.5** | ******* | **9.4** | ****** | 52.3 |  | 12.5 |  | 1.3 |  |
| E96 | 42.5 |  | 785.7 |  | **10.0** | ******* | **50.2** | ****** | 12.5 |  | 2.8 |  | 39.9 |  | 819.3 |  | **9.0** | ******* | 53.0 |  | 12.5 |  | 1.3 |  |
| D874 | **40.3** | ******* | 782.1 |  | **9.1** | ******* | 51.2 |  | 13.2 |  | **2.8*** |  | 39.4 |  | 835.7 |  | **9.0** | ******* | 52.2 |  | 12.7 |  | 1.9 |  |
| D876 | **40.4** | ******* | 786.4 |  | **9.3** | ******* | 51.3 |  | 12.3 |  | 3.0 |  | **38.2** | ****** | 814.6 |  | **8.5** | ******* | 52.6 |  | 13.3 |  | 1.7 |  |
